# Inhibition of HIV-1 infection by TNPO3 depletion is determined by capsid and detectable after viral cDNA enters the nucleus

**DOI:** 10.1186/1742-4690-8-98

**Published:** 2011-12-06

**Authors:** Alberto De Iaco, Jeremy Luban

**Affiliations:** 1 Department of Microbiology and Molecular Medicine, University of Geneva, 1 Rue Michel Servet, CH-1211 Geneva, Switzerland

**Keywords:** HIV-1, capsid, integrase, TNPO3

## Abstract

**Background:**

HIV-1 infects non-dividing cells. This implies that the virus traverses the nuclear pore before it integrates into chromosomal DNA. Recent studies demonstrated that TNPO3 is required for full infectivity of HIV-1. The fact that TNPO3 is a karyopherin suggests that it acts by directly promoting nuclear entry of HIV-1. Some studies support this hypothesis, while others have failed to do so. Additionally, some studies suggest that TNPO3 acts via HIV-1 Integrase (IN), and others indicate that it acts via capsid (CA).

**Results:**

To shed light on the mechanism by which TNPO3 contributes to HIV-1 infection we engineered a panel of twenty-seven single-cycle HIV-1 vectors each bearing a different CA mutation and characterized them for the ability to transduce cells in which TNPO3 had been knocked down (KD). Fourteen CA mutants were relatively TNPO3-independent, as compared to wild-type (WT) HIV-1. Two mutants were more TNPO3-dependent than the WT, and eleven mutants were actually inhibited by TNPO3. The efficiency of the synthesis of viral cDNA, 2-LTR circles, and proviral DNA was then assessed for WT HIV-1 and three select CA mutants. Controls included rescue of TNPO3 KD with non-targetable coding sequence, RT- and IN- mutant viruses, and pharmacologic inhibitors of RT and IN. TNPO3 KD blocked transduction and establishment of proviral DNA by wild-type HIV-1 with no significant effect on the level of 2-LTR circles. PCR results were confirmed by achieving TNPO3 KD using two different methodologies (lentiviral vector and siRNA oligonucleotide transfection); by challenging three different cell types; by using two different challenge viruses, each necessitating different sets of PCR primers; and by pseudotyping virus with VSV G or using HIV-1 Env.

**Conclusion:**

TNPO3 promotes HIV-1 infectivity at a step in the virus life cycle that is detectable after the preintegration complex arrives in the nucleus and CA is the viral determinant for TNPO3 dependence.

## Background

Upon fusing with a target cell membrane, retroviruses release the virion core into the target cell cytoplasm. The core consists of a capsid (CA) protein lattice within which are located the viral genomic RNA, reverse transcriptase (RT), and integrase (IN), among other viral components. RT generates double-stranded DNA using the viral genomic RNA as template, though the exact intracellular location of these reactions, or the structural transformations undergone by the core, are not clear. Recent studies indicate that the CA disassembles (uncoats) in response to reverse transcription [1]. The resulting pre-integration complex (PIC) minimally bears IN and viral cDNA. Immunofluorescence microscopy studies indicate that CA remains associated with a viral structure, perhaps the PIC, that contains the nascent viral cDNA [2] and docks to the nuclear pore [3]. The PIC then gains access to the host nucleus, where IN ligates the viral cDNA into host chromosomal DNA, establishing the provirus. Genetic experiments indicate that CA is critically important for these early steps in the infection cycle that culminate in integration [4-7].

Though the provirus is an essential intermediate in the retroviral replication cycle, not every PIC reaching the nucleus integrates into host cell chromosomal DNA [8]. The termini of the retroviral DNA are recognized by host nuclear factors that join them to produce circular DNAs, which are unable to integrate. Two covalently closed circular forms of retroviral DNA can be found in the nucleus: 1-LTR circles produced by recombination of the long-terminal repeats (LTRs) or 2-LTR circles produced by joining the LTR termini [8,9]. Unlike the permanent provirus, the circular forms are transient, though in some cases they are transcribed and direct protein synthesis [10,11]. Nonetheless, LTR circles provide a valuable indication that the viral cDNA has arrived within the nucleus [12].

The mechanism by which retroviruses gain access to the nucleus is not the same for all retrovirus genera. Lentiviruses such as human immunodeficiency virus type 1 (HIV-1) infect non-dividing cells [13-16], indicating that the lentivirus PIC traverses the nuclear pore. In contrast, mitosis is required for integration by gammaretroviruses like murine leukemia virus (MLV) [15,17].

The HIV-1 PIC is at least ~56 nm [18] and therefore exceeds the 9 nM size-exclusion limit for passive diffusion through the channel of the nuclear pore complex (NPC) [19]. To enter the nucleus then, the PIC likely depends upon an active transport mechanism. Many groups have attempted to determine which viral and cellular factors promote nuclear import of the HIV-1 PIC. Matrix protein (MA) [20], Viral protein R (Vpr) [21], IN [22] and the DNA flap [23] have all been proposed as viral determinants for nuclear entry of the PIC. Experiments using chimeric retroviruses in which HIV-1 CA was replaced with MLV CA, pinpointed CA as a viral determinant for infection of nondividing cells [24]. Moreover, specific CA mutants confer a defect in the ability of HIV-1 to infect nondividing cells [4]. From the standpoint of cellular factors, importin-α [21], importin-β [25,26], importin-7 [27] and transportin-SR2 (TNPO3) [26,28,29], as well as the NPC components NUP153 and NUP358 [26,29-31], have all been implicated in HIV-1 nuclear transport.

TNPO3 is an importin-β-like karyopherin that promotes the nuclear import of serine/arginine-rich splicing factors (SR proteins) [32]. Two genome-wide siRNA screens demonstrated the importance of TNPO3 for HIV-1 replication, and showed that TNPO3 acts early in infection, but after reverse transcription [26,29]. TNPO3 was also identified in a two-hybrid screen for proteins that interact with HIV-1 IN [28], suggesting that TNPO3 promotes nuclear import of the PIC via direct interaction with the IN protein. On the other hand, exploiting the fact that MLV is TNPO3-independent [28], experiments with HIV-1/MLV chimeric viruses mapped the requirement for TNPO3 to CA, not IN [33], and HIV-1 CA mutants have been identified that are TNPO3-independent [6,34]

By examining the effect of TNPO3 on the formation of 2-LTR circles, some studies have suggested that TNPO3 promotes nuclear transport of the HIV-1 PIC, while others have not. The first report on the effect of TNPO3 KD on HIV-1 replication [26] showed a block to provirus formation in TNPO3 KD cells, but omitted experiments showing the level of 2-LTR circles. Two studies reported a decrease in 2-LTR circles in the face of TNPO3 KD [28,35], while two others showed no effect [29,34]. To clarify at which step TNPO3 acts in the HIV-1 life cycle, we examined the fate of viral cDNA under a variety of conditions in cells depleted of TNPO3.

## Results

### TNPO3 knockdown and rescue with non-targetable TNPO3 cDNA

**Figure 1:**
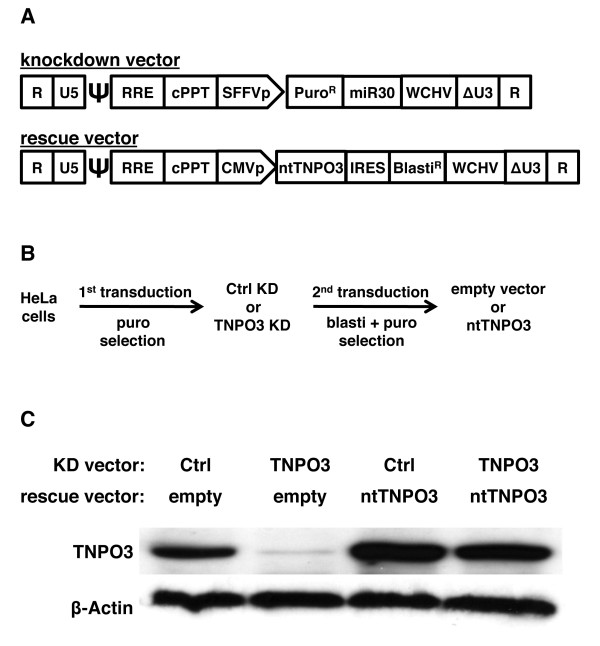
**TNPO3 depletion and rescue with non-targetable TNPO3 cDNA**. **(A) **Schematic representation of the lentiviral vectors used to generate TNPO3 knockdown (KD) and rescue cell lines. **(B) **The sequence of steps used to obtain the four pools of stable cell lines. HeLa cells were transduced with control (Ctrl) KD vector or with TNPO3 KD vector, and selected in pools with 10 μg/ml of puromycin. Each pool of KD cells was then transduced a second time with the rescue vector, either empty or bearing non-targetable TNPO3 cDNA (ntTNPO3), and selected in pools with 10 μg/ml of blasticidin, as well as 1 μg/ml puromycin. **(C) **Steady-state levels of TNPO3 protein in each of the four pools of doubly-tranduced cells. Cell lysate was probed in western blots with anti-TNPO3 antibody (upper panel) and anti-β-actin antibody (lower panel).

To examine the role of TNPO3 in HIV-1 infection, lentiviral vectors were used to generate a panel of HeLa cells with TNPO3 knockdown (KD) and optimal controls (Figure 1A-C). A first round of transduction was conducted with lentiviral KD vectors that encode miR30 microRNAs (miRNAs) engineered to target either TNPO3 mRNA, or firefly luciferase as a control (Figure 1A). The same miRNA-containing, primary transcript of these vectors encodes puromycin N-acetyltransferase [36], so pools of transduced cells were selected in the presence of puromycin. Each of the two pools of KD cells were then transduced a second time with a rescue lentivector expressing a TNPO3 cDNA (ntTNPO3) that bears silent mutations so that it is predicted to be resistant to KD (Figure 1A). The empty version of this vector served as a control. The rescue vector expressed a blasticidin-resistance cassette under the translational control of the encephalomyocarditis virus (EMCV) internal ribosome entry site (IRES). A second round of selection with blasticidin was applied to obtain four pools of cells, each of which was resistant to both puromycin and blasticidin (Figure 1B).

The KD and rescue of TNPO3 in the four pools of cells was verified by western blot (Figure 1C). If anything, the steady-state level of TNPO3 protein in the rescue cells was higher than the endogenous TNPO3 in the starting population of HeLa cells. These four pools of modified HeLa cells were then used for subsequent experiments concerning the effects of TNPO3 on HIV-1 transduction.

### Effect of HIV-1 CA mutants on TNPO3-dependence

**Figure 2:**
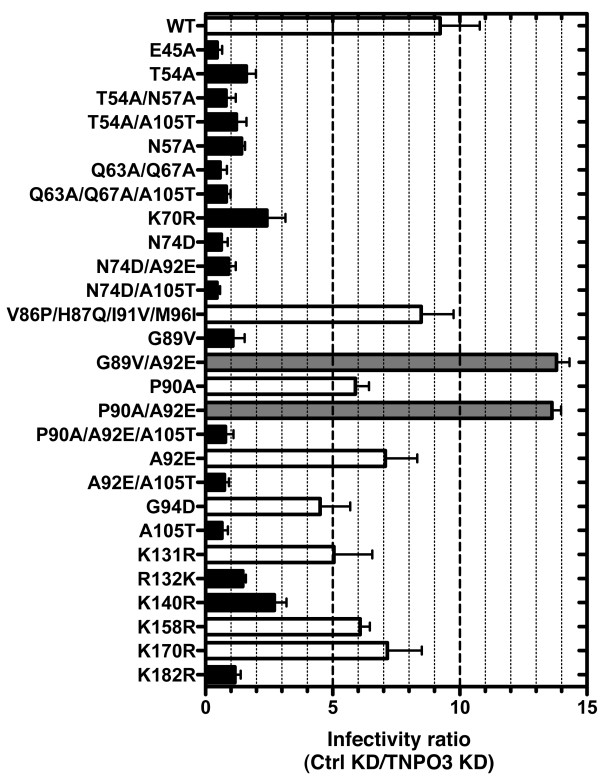
**The effect of TNPO3 KD on the infectivity of HIV-1 CA mutants in HeLa cells**. HeLa control (ctrl) KD cells and TNPO3 KD cells were challenged with a panel of 27 HIV-1-GFP reporter vectors bearing either WT CA or the indicated CA mutants. At 72 hrs the percent GFP^+ ^cells was determined by flow cytometry as an indication of infectivity. The ratio of HIV-1 infectivity in Ctrl KD vs TNPO3 KD cells is shown. Error bars represent ± SEM (n = 3). Black bars indicate mutants that were significantly less sensitive than the WT to TNPO3 KD (p < 0.01, t-test). Gray bars indicate mutants that were significantly more sensitive than the WT to TNPO3 KD (p < 0.01, t-test).

Since previous studies suggest that CA is the viral determinant for HIV-1 replication-dependence on TNPO3 [6,33,34], the infectivity of a panel of 27 HIV-1 CA mutants was compared to that of the wild type (WT), in the context of TNPO3 KD (Figure 2). The CA mutants that were tested here were selected based on previously described phenotypes (Table 1): G89V, P90A, and V86P/H87Q/I91V/M96I are cyclophilin A (CypA)-independent [37-39]; E45A, T54A, T54A/N57A, Q63A/Q67A, K70A, A92E, G94D, and R132K are defective for transduction of cells arrested in the cell-cycle [4,40,41]; E45A, T54A, A92E, and R132K are cyclosporin A (CsA)-dependent [41,42]; G89V, P90A, and A105T each complement the infectivity of CsA-dependent mutants [41,43]; E45A makes a hyper-stable core [44]; T54A, T54A/N57A, and Q63A/Q67A make unstable cores [4,45,46]; E45A, T54A, T54A/N57A, Q63A/Q67A, and N74D have been reported to be TNPO3-independent [6,34]; N74D and P90A are Nup153-independent [30].

**Table 1:** Phenotypes reported for the CA mutants tested in this study.

Phenotypes	CA mutants	References
CypA-independent	G89V, P90A, V86P/H87Q/I91V/M96I	[37-39]

Defective for transduction of cells arrested in the cell-cycle	E45A, T54A, T54A/N57A, Q63A/Q67A, K70A, A92E, G94D, R132K	[4,40,41]

CsA-dependent	E45A, T54A, A92E, G94D, R132K	[41,42]

Complementing the infectivity of CsA-dependent mutants	G89V, P90A, A105T	[41,43]

Hyper-stable core	E45A	[44]

Unstable core	T54A, T54A/N57A, Q63A/Q67A	[4,45,46]

TNPO3-independent	E45A, T54A, T54A/N57A, Q63A/Q67A, N74D, A105S	[6,34]

Nup153-independent	N74D, P90A	[30]

TNPO3 KD and luciferase (Ctrl) KD HeLa cells were challenged with three-part lentiviral vectors encoding a GFP reporter gene and pseudotyped with VSV Glycoprotein (VSV G). Individual vectors bearing each of the CA mutants were compared side-by-side with the WT CA. Three days after challenge with the vectors the extent of transduction was determined by assessing the percent GFP^+ ^cells by flow cytometry. As compared to the control KD cells, transduction of the TNPO3 KD cells by WT HIV-1 was 11-fold lower (Figure 2). CA mutants G89V/A92E and P90A/A92E were significantly more sensitive to TNPO3 depletion than the WT (Figure 2, p < 0.01). Transduction by fourteen of the CA mutants was relatively independent of TNPO3 (Figure 2, p < 0.01). For eleven of the mutants, most prominently E45A, T54A/N57A, Q63A/Q67A, and N74D/A105T, TNPO3 KD increased infectivity from 2 to 4-fold (Figure 2), indicating that TNPO3 inhibits transduction by these viruses. The analysis with this large panel of CA mutants demonstrates that CA is the primary viral determinant for HIV-1 dependence upon TNPO3.

### TNPO3-independent CA mutants cluster on the surface of the CA lattice

**Figure 3:**
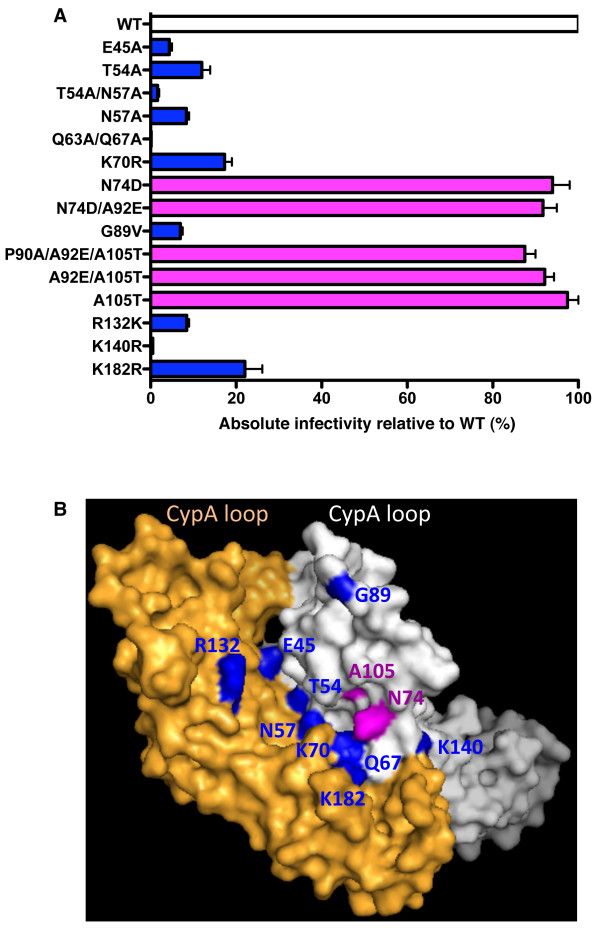
**HIV-1 CA mutants that confer TNPO3-independence localize to the interface between two monomers in the hexameric CA lattice**. **(A) **Absolute infectivity relative to WT virus of TNPO3-independent CA mutants. Data represent one of at least three independent experiments. Error bars represent ± SEM (n = 3). **(B) **Location of CA amino acid residues important for TNPO3-dependence of HIV-1. Space-fill model of a CA dimer extracted from the hexameric structure (PDB: 3H4E). The location of mutants with absolute infectivity similar WT is indicated in magenta. The location of mutants with an absolute infectivity defect are shown in blue; Q63 is in the dimer interface and not visible in this view.

When absolute infectivity relative to WT virus was considered, the TNPO3-independent CA mutants clustered into two groups. N74D, A105T, and most double-mutants involving these residues had absolute infectivity as high as the WT (Figure 3A, purple bars). For E45A, T54A/N57A, Q63A/Q67A, K70A, G89V, R132K, K140R, and K182R, absolute infectivity was reduced as compared with the WT (Figure 3A, dark blue bars).

When viewed on a structural model for the hexameric CA lattice (PDB file #3H4E), the location of most of the CA residues that, when mutated, render HIV-1 TNPO3-independent, is near the point of contact between two CA monomers in the CA hexamer. Figure 3B shows the interface between two CA monomers in isolation from the rest of the CA hexamer model. E45, T54, N57, Q63, Q67, K70, K140 and K182 localize to the actual CA dimer interface. Mutants at these positions have been reported to alter CA stability [4,44-46], a phenotype that may explain the decreased absolute infectivity of these mutants. Amino acids N74 and A105 are located within a pocket adjacent to the CA dimer interface that has been targeted for the development of small molecule inhibitors of HIV-1. One such molecule, PF-3450074 (PF74), destabilizes the CA core [47]. It is interesting that the absolute infectivity for these two TNPO3-independent mutants is not decreased.

### Detailed analysis of selected CA mutants exhibiting TNPO3 phenotypes with minimal fitness cost

**Figure 4:**
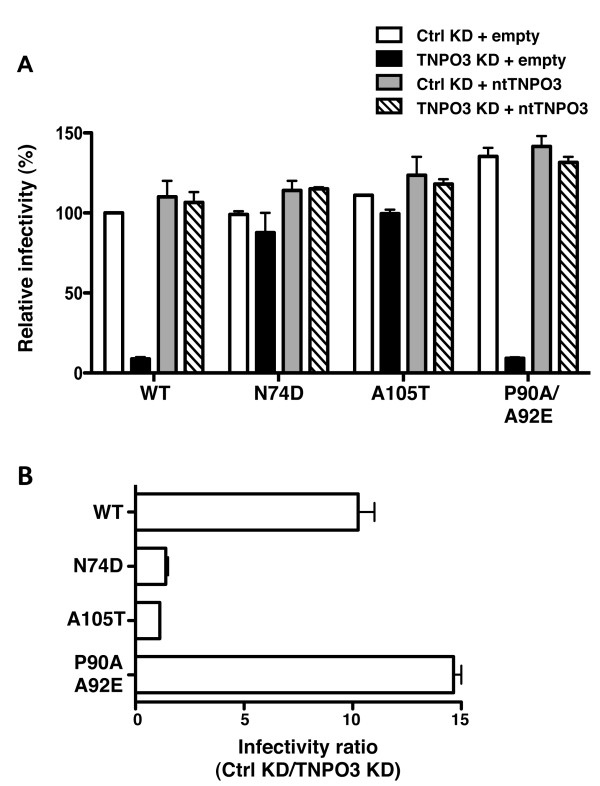
**Infectivity of HIV-1-GFP vectors carrying WT or mutant CA on KD and rescue HeLa cells**. Expression of the GFP reporter gene was checked by flow cytometry 72 hrs after challenge with virus. Infectivity relative to WT is shown in **(A)**. The ratio of the infectivity in TNPO3 KD compared to the control KD cells is shown in **(B)**. Data represent one of at least three independent experiments. Error bars represent ± SEM (n = 3).

Having screened the panel of 27 CA mutants, three mutants with TNPO3 phenotypes, but good absolute infectivity, were selected for more detailed characterization. Each of the four pools of TNPO3 KD and rescue cells described above (Figure 1) were challenged with WT, P90A/A92E, N74D, and A105T CA mutant viruses. Infectivity with virus bearing WT CA was 10-fold lower in TNPO3 KD cells than in Luciferase KD control cells (Figure 4). This defect increased to nearly 15-fold with the P90A/A92E CA mutant virus (p < 0.01). When the ntTNPO3 rescue cells were used as targets, the infectivity of the WT and P90A/A92E viruses was restored to control levels. This indicates that the defect in transduction efficiency by these viruses was due to reduction in TNPO3, and not to off-target effects. In contrast to what was observed with the WT and P90A/A92E viruses, the infectivity of N74D or A105T CA mutant viruses was not decreased by TNPO3 KD (Figure 4). When TNPO3 KD and rescue CD4^+^-HeLa cells were challenged with the same CA mutants in the context of HIV-1 reporter virus bearing the HIV-1 Env glycoprotein, the results were identical to those obtained with VSV G-pseudotyped vector (data not shown).

### The effect of TNPO3 knockdown on HIV-1 replication is detectable after the virus reaches the nucleus

Previous studies concur that TNPO3 depletion results in a block to HIV-1 replication that occurs after reverse transcription has been completed, but before establishment of the provirus [26,28,29,34]. There is disagreement, though, about whether TNPO3 KD changes the levels of the HIV-1 circular cDNAs that serve as a marker for arrival of the viral cDNA into the nucleus [28,29,34,35].

**Figure 5:**
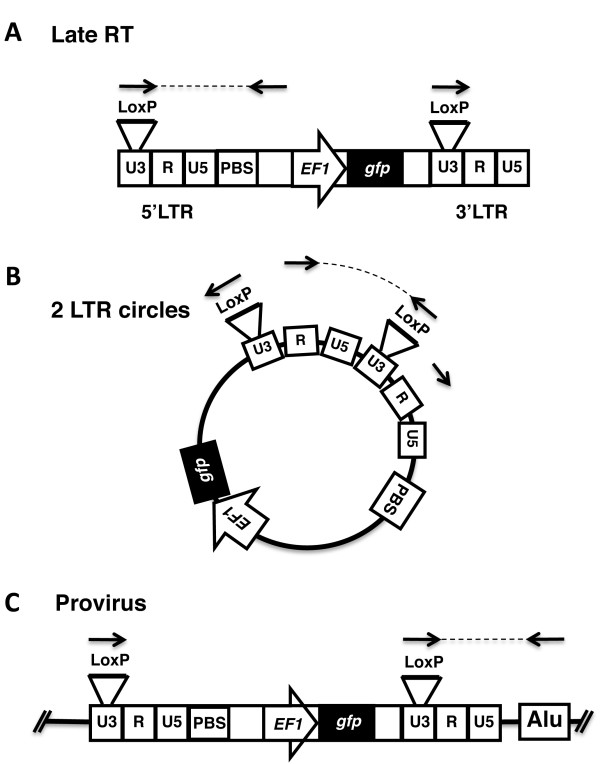
**Strategy for detecting cDNA from HIV-1 reporter virus after challenge of cells that had been previously transduced with HIV-1-based, lentivirus KD and rescue vectors**. Schematic diagram showing methods of detection for HIV-1 late RT products **(A)**, 2-LTR circles **(B) **and provirus **(C) **in KD cell lines. Identification of nascent cDNA is made possible by the presence of a loxP sequence engineered within a region of the 3'LTR U3 that is dispensable for retrotransposition.

**Figure 6:**
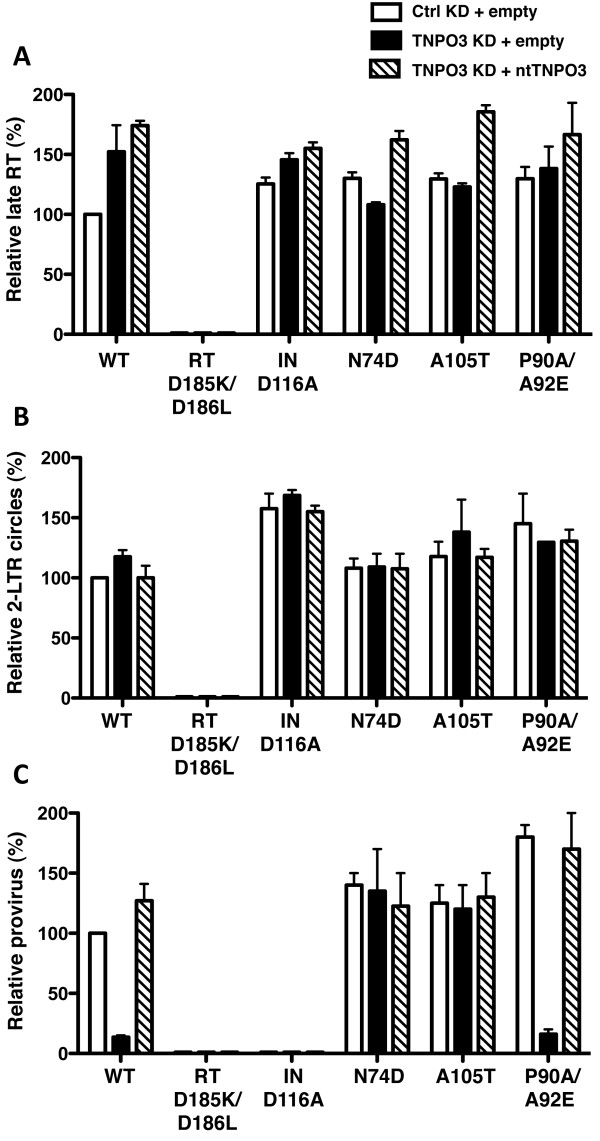
**Effect of TNPO3 on the *de novo *synthesis and fate of HIV-1 cDNA after acute infection**. WT or CA mutant HIV-1 reporter virus were used to challenge the TNPO3 KD and rescue cells, as indicated. 24 hrs later, late RT **(A)**, 2-LTR circles **(B) **and provirus **(C) **were assayed by qPCR. Viruses bearing the enzymatic site mutants RT-D185K/D186L or IN-D116A IN were used as controls for *de novo *reverse transcription and integration, respectively. Data represent one of at least three independent experiments. Error bars represent ± SEM (n = 3).

The fate of the HIV-1 genome during the early phases of infection in the TNPO3-KD and rescue HeLa cells was examined next using quantitative PCR (qPCR). As described above, TNPO3 levels in these cells had been modified by sequential transduction with two different HIV-1 vectors, one for TNPO3 knockdown and one for rescue with ntTNPO3 cDNA (Figure 1). To circumvent the signal that would be generated by these proviruses in PCR reactions using conventional primers to HIV-1 sequences, cells were challenged with pWPTs-GFP, a vector that carries an engineered, 34-nucleotide *loxP *site in the U3 region of the 3'LTR. PCR reactions using a primer complementary to the *loxP *site were able to distinguish pWPTs-GFP-derived signals from those of the two proviruses that were already present in these cells (Figure 5). Cells were challenged for 24 hrs with WT or CA mutant viruses and DNA was extracted. Each sample was subjected to qPCR to assess the amount of late RT products (Figure 6A), 2-LTR circles (Figure 6B), and proviral DNA (Figure 6C). Isogenic HIV-1 viruses bearing previously characterized, inactivating mutations in the catalytic site of RT (D185K/D186K) [48] or in the catalytic site of IN (D116A) [49] were used as controls for the qPCR reactions.

TNPO3 KD had no clear effect on the yield of full-length viral cDNA for any of the viruses tested (Figure 6A). cDNA synthesis by RT mutant D185K/D186K was severely compromised, indicating that the signals observed with the other viruses resulted from *de novo *cDNA synthesis, not from contamination with plasmid DNA used to produce the virus stocks. Similarly, TNPO3 depletion had no detectable effect on the amount of 2-LTR circles formed during infection with WT or CA mutant virus (Figure 6B). The identity of the 2-LTR circles produced by the WT and by each of the mutants was confirmed by sequencing. Finally, TNPO3 KD blocked provirus formation by WT and P90A/A92E mutant viruses (Figure 6C). The magnitude of the defect, 8-fold and 12-fold, respectively, was similar to that of the block in infectivity (compare with Figure 4A). The N74D and A105T mutant viruses, on the other hand, established proviruses independently of TNPO3 (Figure 6C). Provirus formation by the IN mutant D116A was at the level of the background, indicating that a detectable signal in the Alu-PCR reaction requires IN with endonuclease activity.

### TNPO3 KD by transfection of siRNA

**Figure 7:**
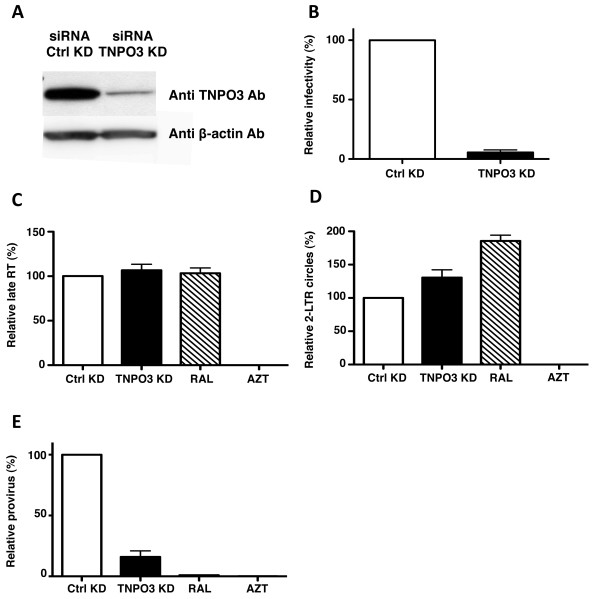
**Effect of TNPO3 KD by transfection of siRNA oligonucleotides on HIV-1 transduction and cDNA synthesis**. HeLa cells were transfected with the indicated siRNAs and challenged 72 hrs later with HIV-1_NL4-3_GFP reporter virus. **(A)**. Cell lysate was probed in western blots with anti-TNPO3 antibody (upper panel) and anti-β-actin antibody (lower panel). **(B) **HIV-1_NL4-3_GFP reporter gene expression was checked by flow cytometry 72 hrs after virus challenge. 24 hrs after challenge with HIV-1_NL4-3_GFP, cell-associated DNA was harvested and processed by qPCR for late RT **(C)**, 2-LTR circles **(D) **and provirus **(E)**. Azidothymidine (AZT) and Raltegravir (RAL) were used to block *de novo *reverse transcription and integration, respectively. Data represent one of at least three independent experiments. Error bars represent ± SEM (n = 3).

To determine if differences in methodology might explain discrepancies between the results reported here and those reported by some other groups [28,35], TNPO3 KD was attempted by transfection of dsRNA oligonucleotides. Reduction in TNPO3 protein in cells transfected with TNPO3-specific siRNA, as compared with cells transfected with the siRNA control, was evident by western blot (Figure 7A). Then, rather than challenge the KD cells with a three-part HIV-1 vector as in the previous experiments, a two-part, VSV G-pseudotyped, pNL4-3-GFP reporter virus was used here. As compared with the cells transfected with the control siRNA, infection of the TNPO3 depleted cells with the pNL4-3-GFP reporter virus was reduced 15-fold (Figure 7B).

Since TNPO3 KD in these cells was accomplished without lentiviral KD vectors, and the challenge virus was an unmodified pNL4-3 that did not possess *loxP *sites, viral cDNA was amplified in qPCR reactions using conventional primers specific for the HIV-1 sequence [50]. As in the previous experiment in Figure 6, TNPO3 KD had no detectable effect on reverse transcription (Figure 7C), or on 2-LTR circle formation (Figure 7D), but provirus formation was severely compromised (Figure 7E). A strong block to integration can be associated with an increased accumulation of DNA circles [29]. TNPO3 KD was indeed associated with a 30% increase in 2-LTR circles, as compared with an 80% increase in the presence of the IN strand-transfer inhibitor Raltegravir. The products in these qPCR experiments were detected with SYBR green but identical results were obtained when products were detected with TaqMan probes (data not shown), as previously described [50]. Taken together, these results indicate that the block to HIV-1 replication in the face of TNPO3 KD is detectable after the virus reaches the nucleus.

### The effect of TNPO3 KD on HIV-1 transduction of CD4^+ ^T cells

**Figure 8:**
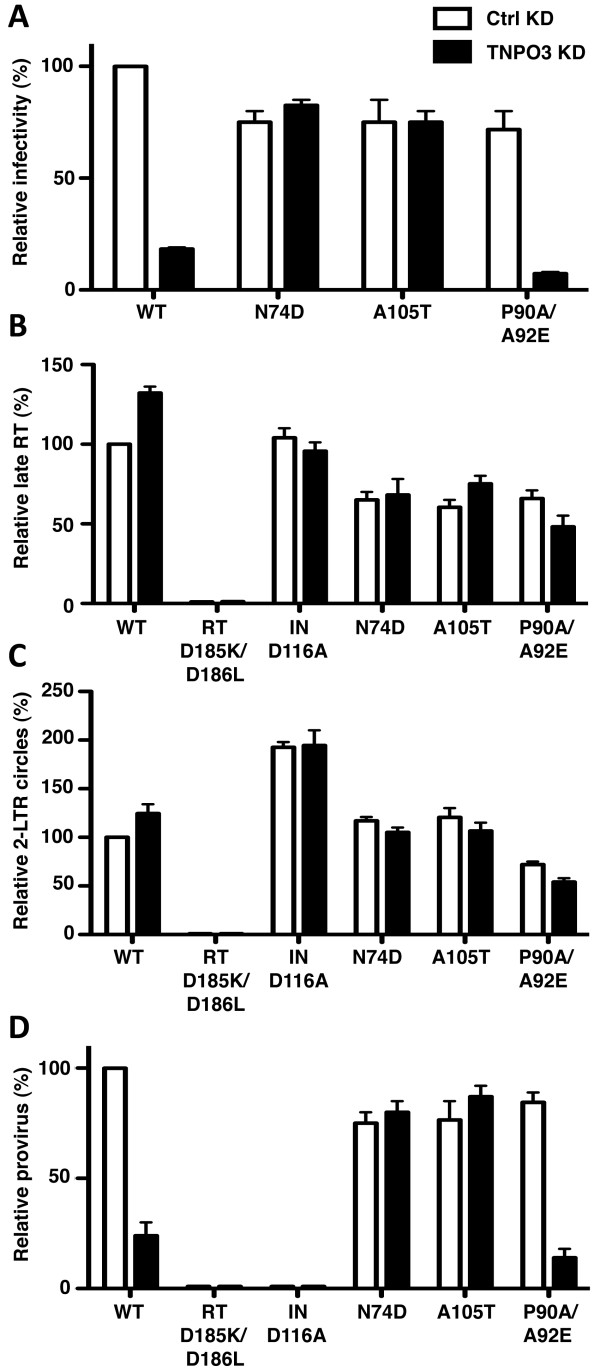
**Effect of TNPO3 KD on HIV-1 infection of CD4^+ ^T cells**. Jurkat T cells, modified with TNPO3 KD or control KD lentiviruses, were challenged with WT or CA mutant HIV-1 viral vectors, as indicated. **(A) **GFP reporter gene expression was checked by flow cytometry 72 hrs after HIV-1 challenge. Late RT **(B)**, 2-LTR circles **(C) **and provirus **(D) **were assayed by qPCR 24 hrs after infection. Viruses bearing the enzymatic site mutants RT-D185K/D186L or IN-D116A IN were used as controls for *de novo *reverse transcription and integration, respectively. Data represent one of at least three independent experiments. Error bars represent ± SEM (n = 3).

In the previous experiments, the effect of TNPO3 depletion was assessed using HeLa cervical cancer cells. Identical results were obtained when the lentiviral KD and rescue vectors were used in TE671 cells (data not shown). Since CD4^+ ^T lymphocytes are the primary cellular target of HIV-1, TNPO3 KD Jurkat T cells were also generated with the lentiviral KD vectors. Infectivity with virus bearing WT CA was 5-fold lower in TNPO3 KD cells than in control KD cells (Figure 8A). P90A/A92E CA mutant virus showed higher inhibition compared to WT virus, to nearly 10-fold. N74D and A105T were not inhibited by TNPO3 KD in Jurkat T cells (Figure 8A).

The early steps of HIV-1 infection in TNPO3 KD Jurkat cells were then characterized using the qPCR method described in Figure 5. Jurkat T cells were challenged for 24 hrs with WT or CA mutant viruses and DNA was extracted. Each sample was then subjected to qPCR to assess the amount of late RT products (Figure 8B), 2-LTR circles (Figure 8C), and proviral DNA (Figure 8D). RT and IN mutant viruses were used as controls for the qPCR reactions.

TNPO3 KD in Jurkat cells had no effect on the amount of full-length viral cDNA produced by WT or CA mutant viruses (Figure 8B). Similarly, no significant effect was detected in the amount of 2-LTR circles produced by WT or CA mutant viruses infecting Jurkat depleted of TNPO3 (Figure 8C). IN mutant D116A showed a 2-fold increase in the amount of 2-LTR circles synthesized. Finally, TNPO3 depletion blocked integration of WT and P90A/A92E mutant viruses, with a magnitude of defect similar to that of the block in infectivity (Figure 8D).

## Discussion

### CA determines HIV-1 dependence on TNPO3

A previous study demonstrated that CA, and not IN, is the viral determinant of TNPO3-dependence, at least when these components of HIV-1 were exchanged with those of the TNPO3-independent virus MLV [33]. Though the results of that study were clear, interpretation was complicated by the fact that HIV-1/MLV chimeras are severely attenuated in terms of absolute infectivity. Subsequent studies identified individual CA residues that confer TNPO3-independence to HIV-1 [6,34]. Now we show that TNPO3 KD caused less than 2-fold reduction in infectivity for 18 CA mutants, among which five had normal absolute infectivity. Additionally, two CA mutants were identified that are hypersensitive to TNPO3 KD. From the analysis of 27 CA mutants presented here, then, the importance of CA for HIV-1 dependence on TNPO3 is unequivocal. While our studies do not directly address the importance of IN for TNPO3-dependence, one would need to invoke a rather complex model to reconcile the data reported here with one in which HIV-1 IN function requires TNPO3.

### CA mutants, CA lattice stability, and host factors

A significant fraction of the TNPO3-independent CA mutants have been reported previously to have effects on CA stability [4,44-46]. Amino acids E45, T54, N57, Q63, Q67, K70, K140 and K182 are all located at the interface between CA dimers in the CA lattice (Figure 3B). It is easy to imagine how changes in these positions would alter contact between CA monomers, resulting in a CA lattice with decreased stability. This would also explain the decreased absolute infectivity of these mutants (Figure 3A).

Among the TNPO3-independent mutants, N74 and A105 localize to a pocket on the CA surface that is near the dimer interface. This pocket is the target of a drug that destabilizes the CA core [47]. The N74D and A105T mutants might also decrease core stability, like the CA mutants at the dimer interface, but to our knowledge this has not been demonstrated. Given that the absolute infectivity of N74D and A105T is normal, it seems unlikely that the major effect of these mutants would be to decrease core stability.

Mutants N74D and A105T might perturb binding to the CA pocket of a cellular factor that alters CA stability. TNPO3 itself has been reported to bind to CA [34] and it potentially destabilizes the capsid lattice to facilitate uncoating. Alternatively, TNPO3 might regulate another cellular factor that binds to CA. A good candidate for this putative factor would be cleavage and polyadenylation factor 6 (CPSF6), an SR-protein involved in the processing of mRNA [51]. A C-terminal truncated form of CPSF6 (CPSF6-358) inhibits HIV-1 infection and the TNPO3-independent CA mutant viruses N74D, Q63A/Q67A and E45A are resistant to CPSF6-358 restriction [6]. TNPO3 is a β-karyopherin that mediates nuclear import of SR-proteins. The CPSF6-358 truncation removes the SR-rich domain, suggesting that TNPO3 regulates HIV-1 by controlling CPSF6 localization. How CPSF6-358 would inhibit HIV-1 infection is unclear, but it might inhibit the virus by altering CA stability.

CypA, a peptidylprolyl isomerase that directly binds HIV-1 CA [52], also seems to act on the stability of the CA core during HIV-1 infection [52,53]. In fact, the CsA-dependent CA mutant A92E has decreased core stability, which is restored if CA-CypA binding is blocked [53]. The TNPO3-independent CA mutant A105T rescues a conditional infectivity defect of the A92E CA mutant virus [41], without altering the binding of CA to CypA (De Iaco and Luban, unpublished data).

### The effect of TNPO3 on HIV-1 is first detectable after the viral cDNA enters the nucleus

Since TNPO3 is a karyopherin that binds HIV-1 IN, most studies have attempted to demonstrate a role for TNPO3 in nuclear import of the HIV-1 PIC. Some of them have been able to show it, some others not [26,28,29,34,35]. As a result of this controversy, we examined the role of TNPO3 in detail, using a range of methodologies. As part of this analysis we developed tools for discriminating viral cDNA generated by a reporter virus in cells that had already been transduced with KD and rescue lentiviral vectors; this new assay will prove to be valuable for future studies of host factors of importance to HIV-1.

Using a range of approaches we demonstrated that TNPO3 has no detectable effect on the amount of 2-LTR circles produced during HIV-1 infection. Given the limitations of this indirect assay for nuclear import of the PIC, our results suggest that TNPO3 acts on HIV-1 after the PIC arrives in the nucleus. It remains a possibility, though, that TNPO3 acts in the cytoplasm to promote maturation of the PIC via some unknown mechanism. One group reported that TNPO3 is required for nuclear import of PICs that were visualized directly by GFP-labeled IN [28] but the functional significance of these labeled complexes has not been established.

According to generally accepted models of the HIV-1 replication cycle, HIV-1 CA uncoats in the cytoplasm to permit reverse transcription, or perhaps uncoats in response to reverse transcription [1]. Given our conclusion that TNPO3 acts in the nucleus to promote HIV-1 infectivity, how would this fact be reconciled with the importance of CA stability and uncoating for TNPO3-dependence? It may be that some CA protein travels to the nucleus with the PIC, and, as recently reported, that TNPO3 promotes HIV-1 infection by stripping the residual CA associated with the PIC and exporting this extracted CA to the cytosol [34]; this would free the viral cDNA for more efficient integration.

## Methods

### Cell lines, tissue culture, and drugs

293T, TE671 and HeLa cells were grown in Dulbecco's modified Eagle medium (D-MEM) (Invitrogen) supplemented with 10% fetal bovine serum (FBS) (PAA), 20 mM L-glutamine, 1000 U/ml penicillin, and 1000 mg/ml Streptomycin (GIBCO). Jurkat CD4^+ ^T cells were grown in RPMI-1640 (Invitrogen) supplemented with 10% FBS. Azidothymidine (AZT, NIBSC, UK) and Raltegravir (RAL, Santa Cruz) were used at concentrations of 25 μM and 10 μM, respectively.

### Plasmids

pWPTs-GFP is an HIV-1-based transfer vector with EGFP expression under the control of the EF1α promoter [54]. p8.9NdSB is a minimal HIV-1 packaging plasmid for *gag *and *pol *expression [55]. pMD2-G encodes the vesicular stomatitis virus G protein (VSV-G) [56]. pNL4-3.GFP.E^- ^[57] bears HIV-1 proviral sequence with an *env*-inactivating mutation and EGFP in place of *nef*. pAPM is an HIV-1 based knockdown vector in which a single transcript driven by the spleen focus-forming virus (SFFV) LTR contains a miR30 framework modified to target a gene of interest and the puromycin N-acetyltransferase gene [36]. pAIB is an HIV-1 based transfer vector expressing the protein of interest from the SFFV LTR followed by the encephalomyocarditis virus (EMCV) internal ribosome entry site (IRES) cassette and blasticidin-S-deaminase, drug resistance cassette [36]. pETSUMO-hTNPO3 bearing the human TNPO3 cDNA was generously provided by Peter Cherepanov.

### Knockdown Vector Cloning

Using the RNAi Central web-based shRNA design program (http://cancan.cshl.edu/RNAi_central/RNAi.cgi?type=shRNA - lab of Dr. Gregory Hannon, Cold Spring Harbor Laboratory), we designed three miRNA-based shRNA targeting sequences against the TNPO3 transcript. Three 97-mer oligonucleotides were synthesized and PAGE purified: ts1, 5'-TGCTGTTGACAGTGAGCGACGCCAGAATCTGTGGACTCAATAGTGAAGCCACAGATGTATTGAGTCCACAGATTCTGGCGCTGCCTACTGCCTCGGA-3'; ts69, 5'-TGCTGTTGACAGTGAGCGCCCTCAATATGAGGTAGTAGAATAGTGAAGCCACAGATGTATTCTACTACCTCATATTGAGGATGCCTACTGCCTCGGA-3'; ts72, 5'-TGCTGTTGACAGTGAGCGCGGACAGTAACTTCATGGCTAATAGTGAAGCCACAGATGTATTAGCCATGAAGTTACTGTCCATGCCTACTGCCTCGGA-3'. The 97-mer oligonucleotides were then amplified by PCR using the following primers: miR-30 XhoI 5': 5'-AAGGCTCGAGAAGGTATATTGCTGTTGACAGTGAG-3' and miR-30 EcoRI 3': 5'-AGCCCCTTGAATTCCGAGGCAGTAGGCA-3'. The PCR reaction was carried out with AccuPrime Pfx SuperMix, 1 mol⁄L Betaine(Sigma-Aldrich), 0.4 μmol⁄L each primer, and 100 ng 97-mer oligonucleotide template. The PCR product was column purified, digested with XhoI and EcoRI, and ligated in pAPM to create the pAPM-TNPO3 constructs (ts1, ts69 and ts72). The function of TNPO3 KD vectors (ts1, ts69 and ts72) was checked by generating stable, puromycin-selected HeLa cells. Efficiency of the TNPO3 KDs was analyzed by looking at the expression levels of TNPO3. Both ts69 and ts72 strongly inhibited the expression of TNPO3, but ts72 was selected for most experiments since of the three constructs it was the most potent.

### Mutagenesis

Overlapping PCR was used to generate mutations in the CA, RT and IN coding sequences of p8.9NdSB using the mutagenic oligonucleotides in Table 2.

**Table 2:** Oligonucleotides used for cloning in this study.

Primer name	Primer sequence
E45A fwd	5'-CAGCATTATCAGCTGGAGCCACCCC-3'

E45A rev	5'-GGGGTGGCTCCAGCTGATAATGCTG-3'

T54A fwd	5'-CCACAAGATTTAAACGCCATGCTAAACACAGTGG-3'

T54A rev	5'-CCACTGTGTTTAGCATGGCGTTTAAATCTTGTGG-3'

T54A/N57A fwd	5'- CCACAAGATTTAAACGCCATGCTAGCCACAGTGGGGGGAC-3'

T54A/N57A rev	5'-GTCCCCCCACTGTGGCTAGCATGGCGTTTAAATCTTGTGG-3'

N57A fwd	5'-CACCATGCTAGCCACAGTGGGGGGAC-3'

N57A rev	5'-GTCCCCCCACTGTGGCTAGCATGGTG-3'

Q63A/Q67A fwd	5'-GTGGGGGGACATGCAGCAGCCATGGCAATGTTAAAAGAGAC-3'

Q63A/Q67A rev	5'-GTCTCTTTTAACATTGCCATGGCTGCTGCATGTCCCCCCAC-3'

K70R fwd	5'-GCAGCCATGCAAATGTTAAGAGAGACCATCAATGAGGAAG-3'

K70R rev	5'-CTTCCTCATTGATGGTCTCTCTTAACATTTGCATGGCTGC-3'

N74D fwd	5'-GAGACCATCGATGAGGAAGCTGCAGAATGG-3'

N74D rev	5'-CCATTCTGCAGCTTCCTCATCGATGGTCTC-3'

G89V fwd	5'-CCAGTGCATGCAGTGCCTATTGCACCAGGCCAG-3'

G89V rev	5'-CTGGCCTGGTGCAATAGGCACTGCATGCACTGG-3'

G89V/A92E fwd	5'-CCAGTGCATGCAGTGCCAATTGAGCCAGGCCAGATGAG-3'

G89V/A92E rev	5'-CTCATCTGGCCTGGCTCAATTGGCACTGCATGCACTGG-3'

P90A fwd	5'-CCAGTGCATGCAGGTGCCATTGCACCAGGCCAGATG-3'

P90A rev	5'-CATCTGGCCTGGTGCAATGGCACCTGCATGCACTGG-3'

P90A/A92E fwd	5'-CCAGTGCATGCAGGCGCAATTGAGCCAGGCCAGATG-3'

P90A/A92E rev	5'-CATCTGGCCTGGCTCAATTGCGCCTGCATGCACTGG-3'

A92E fwd	5'-GTGCATGCAGGGCCAATTGAGCCAGGCCAGATG-3'

A92E rev	5'-CATCTGGCCTGGCTCAATTGGCCCTGCATGCAC-3'

G94D fwd	5'-GTGCATGCAGGGCCCATTGCACCAGACCAGATGAGAGAACC-3'

G94D rev	5'-GGTTCTCTCATCTGGTCTGGTGCAATGGGCCCTGCATGCAC-3'

A105T fwd	5'-GGAAGTGACATAACAGGAACTACTAGTACC-3'

A105T rev	5'-GGTACTAGTAGTTCCTGCTATGTCACTTCC-3'

K131R fwd	5'-CCAGTAGGAGAAATCTATAGAAGATGGATAATCCTGG-3'

K131R rev	5'-CCAGGATTATCCATCTTCTATAGATTTCTCCTACTGG-3'

R132K fwd	5'-GGAGAAATCTATAAAAAATGGATAATCCTGGG-3'

R132K rev	5'-CCCAGGATTATCCATTTTTTATAGATTTCTCC-3'

K140R fwd	5'-GGATAATCCTGGGATTAAATAGAATAGTAAGAATGTATAGC-3'

K140R rev	5'-GCTATACATTCTTACTATTCTATTTAATCCCAGGATTATCC-3'

K158R fwd	5'-GACATAAGACAAGGACCAAGGGAACCCTTTAGAGACTATG-3'

K158R rev	5'-CATAGTCTCTAAAGGGTTCCCTTGGTCCTTGTCTTATGTC-3'

K170R fwd	5'-CTATGTAGACCGATTCTATAGAACTCTAAGAGCCGAGC-3'

K170R rev	5'-GCTCGGCTCTTAGAGTTCTATAGAATCGGTCTACATAG-3'

K182R fwd	5'-CAAGCTTCACAAGAGGTAAGAAATTGGATGACAGAAACC-3'

K182R rev	5'-GGTTTCTGTCATCCAATTTCTTACCTCTTGTGAAGCTTG-3'

NotI fwd	5'-CCTTGGCTTCTTATGCGACGG-3'

SpeI rev	5'-GTCCAGAATGCTGGTAGGGC-3'

SpeI fwd	5'-GCTGCAGAATGGGATAGAGTGC-3'

ApaI rev	5'-GTGGGAAGGCCAGATCTTCC-3'

D185K/D186K fwd	5'-GACATAGTCATCTATCAATACATGAAGCTTTTGTATGTAGGATCTGACT TAG-3'

D185K/D186K rev	5'-CTAAGTCAGATCCTACATACAAAAGCTTCATGTATTGATAGATGACTA TGTC-3'

EcoRV fwd	5'-GTTCCCTTAGATAAAGACTTCAGG-3'

AgeI rev	5'-GATATGTCCATTGGCCTTGC-3'

D116A fwd	5'-CAGTAAAAACAGTACATACAGCCAATGGCAGCAATTTCACC-3'

D116A rev	5'-GGTGAAATTGCTGCCATTGGCTGTATGTACTGTTTTTACTG-3'

AgeI fwd	5'-CCAAAGCACTAACAGAAGTAGTACC-3'

EcoRI rev	5'-CGAGTAACGCCTATTCTGCTATG-3'

ntTNPO3 fwd	5'-GTTGGTTTAACTTGGGAGTTTTAGATTCGAATTTTATGGCTAACAATAA ATTACTAGCAC-3'

ntTNPO3 rev	5'-GTGCTAGTAATTTATTGTTAGCCATAAAATTCGAATCTAAAACTCCCAA GTTAAACCAAC-3'

AflII fwd	5'-TGTGTGCTTAAGCTCGAGTGGTGGCATGGAAGGAGCAAAGC-3'

KpnI rev	5-GTAAGCTTTGAAGATGCCATG-3'

CMV fwd	5'- TGTGTGTTAATTAAGCGTTGACATTGATTATTGACTAG-3'

CMV rev	5'- CACACACTCGAGCCCTTGTCATCGTCGTCCTTGTAGTCCATGTTTAAA CGCTAGCCAGCTTG-3'

For the CA mutants E45A, T54A, T54A/N57A, N57A, Q63A/Q67A, K70R, N74D, G89V, G89V/A92E, P90A, P90A/A92E, A92E, G94D and A105T, a PCR product of 957 bp was amplified using the external oligonucleotides NotI fwd and SpeI rev (Table 2). The PCR products were digested with NotI and SpeI and ligated to p8.9NdSB cut with the same enzymes. For the CA mutants K131R, R132K, K140R, K158R, K170R and K182R, a PCR product of 499 bp was amplified using the external oligonucleotides SpeI fwd and ApaI rev (Table 2). The PCR products were digested with SpeI and ApaI and ligated to p8.9NdSB cut with the same enzymes. CA mutant V86P/H87Q/I91V/M96I was previously cloned [58].

For the RT mutant D185K/D186K a PCR product of 667 bp was amplified using the external oligonucleotides EcoRV fwd and AgeI rev (Table 2). The PCR product was digested with EcoRV and AgeI and ligated to p8.9NdSB cut with the same enzymes. For IN mutant D116A a PCR product of 1767 bp was amplified using the external oligonucleotides AgeI fwd and EcoRI rev (Table 2). The PCR product was digested with AgeI and EcoRI and ligated to p8.9NdSB cut with the same enzymes. All mutations were confirmed by sequencing.

To render TNPO3 expression resistant to TNPO3 KD, silent mutations were introduced into the TNPO3 cDNA by overlapping PCR using the oligonucleotides ntTNPO3 fwd; ntTNPO3 rev (Table 2) This was cloned into pAIB [36]. The ntTNPO3 sequence was then confirmed by DNA sequencing. The SFFV promoter driving ntTNPO3 transcription in the pAIB vector was then replaced with the CMVie promoter from pcDNA3.1- to obtain a higher level of expression of TNPO3 expression in HeLa cells. At the same time an HA-tag was introduced at the N-terminus of the protein. The primers used were CMV fwd and CMV rev (Table 2)

### Production of viruses and vectors

Viruses and minimal vectors were produced by transfection of 293T cells using Polyethylenimine (PEI) (Sigma, Inc). 4 × 10^6 ^cells per plate were seeded in 10 cm plates, one day prior to transfection. The DNA for the transfection was mixed with 50 μl of PEI (1 mg/ml) in 1 ml of DMEM without serum for 20 min at room temperature. 6 hrs after transfection the transfection medium was replaced with fresh target-cell medium. 48 hrs after transfection the supernatant was collected, centrifuged at 1200 × *rpm *for 5 min, filtered through a sterile 0.45 μm syringe filter (Millipore), and stored in 1 mL aliquots at -80°C. When comparing viruses or vectors, samples were normalized by RT activity present in the viral supernatant using the PCR-based assay described below. For three-part vector systems, the following DNA ratio was used: 3 parts transfer vector: 2 parts packaging plasmid: 1 part envelope. For two-part virus systems a 4:1 ratio was used (7 parts env^- ^virus: 1 part envelope)

### Reverse transcriptase assay (SGPERT)

Reverse transcriptase (RT) activity in the supernatants was quantified using a Sybr green I-based real-time PCR enhanced RT assay (SGPER) that possesses both high sensitivity and an extraordinary dynamic range. The assay is a modified version of one described previously [59]. Briefly, virions in cell-free supernatant were disrupted by adding an equal volume of SGPERT lysis buffer containing 0.25% Triton X-100, 50 mM KCl, 100 mM TrisHCl pH7.4, 0.4 U/μl RNase inhibitor (RiboLock, MBI Fermentas). Lysed virions were used for reverse transcription of MS2 RNA template (Roche) [60]. Quantification of reverse transcribed products was carried out in a CFX96 thermal cycler (Biorad) using Sybr-Green I, hotstart Taq and reaction buffer (Fermentas), and an MS2 primer set as already described [60]. A standard curve was obtained using known concentrations (expressed in functional units) of recombinant HIV-1 RT (Ambion).

### Generation of TNPO3 KD cells and rescue of the TNPO3 protein

To generate stable microRNA-based shRNA KDs, HeLa cells were transduced with pAPM microRNA-based shRNA vectors [36] targeting either control or TNPO3 mRNA (ts1, ts69, and ts72). Two days after transduction, the cells were selected with 10 μg⁄mL puromycin dihydrochloride. To generate the TNPO3 rescue cells, HeLa TNPO3 KD and control KD cells were transduced with the pAIB-CMV expression vector, either empty or encoding the ntTNPO3. 2 days after transduction, the cells were selected with 10 μg⁄mL blasticidin for 3 days and assayed for KD and protein rescue by SDS-PAGE⁄western blot.

For transient siRNA, 100 nM of Gene Solution siRNA (Qiagen) targeting TNPO3 was complexed with 5 μl of Lipofectamine RNAiMAX (Invitrogen), following the manufacturer's instruction, and added to the cells. KD was assessed by SDS-PAGE/western blot 72 h after transfection.

### Quantitative PCR for viral cDNA

Total DNA was extracted from 4 × 10^6 ^cells using the DNeasy Blood & Tissue Kit (Qiagen), following the manufacturer's instructions.

Quantitative PCR for pWPTs-GFP full-length linear cDNA and 2-LTR circles were detected with Sybr green (Invitrogen), while Alu-PCR used a TaqMan probe. The primers used for the detection of late RT products were: pWPT J1B fwd and pWPT J2 rev (Table 3). The primers used for the detection of 2-LTR circles were: R2 fwd and LoxP2 rev (Table 3). The primers used for the analysis of the proviruses were: pWPT J1B fwd, SB704 rev and MH603 probe [50] (Table 3).

**Table 3:** Oligonucleotides used for pWPTs-GFP quantitative PCR.

	Primer name	Primer sequence
**Late RT**	pWPT J1B fwd	5'-GCATACATTATACGAAGTTATGCTGC-3'
	
	pWPT J2 rev	5'-GCCGTGCGCGCTTCAGCAAGC-3'

**2-LTR**	R2 fwd	5'-TGGGAGCTCTCTGGCTAACTAG-3'
	
	LoxP2 rev	5'-GTGAATTGATCCCATCTTGTC-3'

**Alu PCR**	pWPT J1B fwd	5'-GCATACATTATACGAAGTTATGCTGC-3'
	
	SB704 rev	5'-TGCTGGGATTACAGGCGTGAG-3'
	
	MH603 probe	5'-(FAM)-ACACTACTTGAAGCACTCAAGGCAAGCTTT-(TAMRA)-3'

Late RT and 2-LTR circle PCR reaction mixes contained 1× Sybr green mix (10 mM Tris pH 8.3, 10 mM KCl, 2.5 mM NH_4_SO_4_, 5 mM MgCl_2_, 0.1 mg/ml BSA, 0.2 mM dNTPs, 1× Sybr green), 300 nM each primer, 100 to 200 ng of template DNA, and 1 μl of Hot Start Taq Polymerase (Promega) in a volume of 20 μl. Alu PCR reaction mix contained 1× TaqMan Universal Master Mix (Applied Biosystems), 50 nM pWPT J1B fwd primer, 900 nM SB704 rev primer, 100 nM MH603 probe and 100 to 200 ng of template DNA in a volume of 20 μl.

For late RT and 2-LTR circle analysis, after initial incubation at 95°C for 2 min to activate the Hot Start Taq Polymerase, 40 cycles of amplification and acquisition were carried out at 95°C for 6 s, followed by 10 s at 55°C, 30 s at 72°C and 6 s at 80°C. For Alu PCR, after an initial incubation at 95°C for 10 min, 40 cycles of amplification were carried out at 95°C for 15 s followed by 1 min and 30 s at 60°C. qPCR reactions were made using the CFX96™ thermal cycler (Biorad).

qPCR for NL4.3 GFP E- full-length linear cDNA, 2-LTR circles and Alu PCR was performed as described previously [50] (Table 4).

**Table 4:** Oligonucleotides used for NL4.3 GFP E- quantitative PCR (Butler 2001).

	Primer name	Primer sequence
**Late RT**	MH531	5'-GCATACATTATACGAAGTTATGCTGC-3'
	
	MH532	5'-GCCGTGCGCGCTTCAGCAAGC-3'
	
	LRT-P	5'-(FAM)-CAGTGGCGCCCGAACAGGGA-(TAMRA)-3'

**2-LTR**	MH535	5'-AACTAGGGAACCCACTGCTTAAG-3'
	
	MH536	5'-TCCACAGATCAAGGATATCTTGTC-3'
	
	MH603 probe	5'-(FAM)-ACACTACTTGAAGCACTCAAGGCAAGCTTT-(TAMRA)-3'

**Alu PCR**	MH535	5'-AACTAGGGAACCCACTGCTTAAG-3'
	
	SB704 rev	5'-TGCTGGGATTACAGGCGTGAG-3'
	
	MH603 probe	5'-(FAM)-ACACTACTTGAAGCACTCAAGGCAAGCTTT-(TAMRA)-3'

Where indicated, cells were treated with AZT or RAL, 1 hr before infection at concentrations of 25 μM and 10 μM, respectively.

### Western blot analysis

For western blot analysis we used rabbit anti-TNPO3 antibody (ab71388, abcam) and mouse anti-actin antibody (Sigma). The secondary antibodies were HRP-linked donkey anti-rabbit IgG and HRP-linked sheep anti-mouse IgG (GE Healthcare Life Sciences).

## List of abbreviations

CA: Capsid;IN: Integrase; RT: reverse transcriptase; KD: knockdown; WT: wild-type; PIC: preintegration complex; LTR: long terminal repeat; HIV-1: human immunodeficiency virus type 1; MLV: Moloney murine leukemia virus; NPC: nuclear pore complex; CypA: cyclophilin A; CsA: cyclosporine; CPSF6: cleavage and polyadenylation factor 6; SFFV: spleen focus forming virus; IRES: internal ribosome entry site; CMV: cytomegalovirus.

## Competing interests

The authors declare that they have no competing interests.

## Authors' contributions

AD and JL conceived and designed the experiments, analyzed the data, and wrote the paper. AD performed the experiments. Both authors read and approved the final manuscript.

## Acknowledgements

We would like to thank Massimo Pizzato and Federico Santoni for technical assistance, and Peter Cherepanov for reagents. This work was supported by NIH grant RO1AI59159 and Swiss National Science Foundation grant 3100A0-128655 to J.L. The funders had no role in study design; in the collection, analysis, and interpretation of data; in the writing of the manuscript; or in the decision to submit the manuscript for publication.
